# Participants’ expectations and experiences with periodic health examinations in Austria - a qualitative study

**DOI:** 10.1186/s12913-018-3640-6

**Published:** 2018-10-30

**Authors:** Isolde Sommer, Viktoria Titscher, Gerald Gartlehner

**Affiliations:** 1 Department for Evidence-based Medicine and Clinical Epidemiology, University of Continuing Education (Danube University Krems), Dr.-Karl-Dorrek-Straße 30, 3500 Krems, Austria; 2 RTI-UNC Evidence-based Practice Center, Research Triangle Institute International, 3040 East Cornwallis Road, P.O. Box 12194, Research Triangle Park, Raleigh, North Carolina 27709-2194 USA

**Keywords:** Periodic health examination, Health check, Screening, Focus groups, Qualitative study, Motivations, Expectations, Satisfaction

## Abstract

**Background:**

The engagement of citizens in the development of evidence-based screening programs is internationally supported. The aim of our research was to explore the motivations and reasons of adult citizens in Austria for attending periodic health examinations (PHE) as well as their satisfaction with the way PHE are organized.

**Methods:**

We conducted three focus groups with a random sample of previous attenders of PHE. Participants were stratified by age, gender, and education. The discussions were recorded, transcribed, and analyzed using a thematic analysis approach.

**Results:**

Main motivations of attenders (*n* = 30) were to detect diseases early, to prevent suffering, and to live a long, healthy life. They believed that PHE work as an incentive of health behavior change. As possible reasons not to attend PHE, participants mentioned lack of awareness, time constraints, unpleasant prior experiences, and fear of harm or negative consequences. They wanted the range of examinations to be selected based on individual risks and to be more comprehensive. Some participants expressed frustration with the lack of time doctors dedicated to the examination or discussion of the results. Throughout the discussion, participants realized there is a great diversity among doctors in the quality of health examinations and how content is delivered.

**Conclusion:**

The study showed that attenders of PHE have high expectations concerning the beneficial outcomes of PHE. They requested a comprehensive and individualized program that does not reflect the scientific evidence from effectiveness studies of PHE. These findings indicate serious shortcomings in the communication of benefits and harms of screening interventions and highlight the need for a more proactive communication about aims and content of the program.

## Background

Periodic health examinations (PHE) are an essential part of clinical prevention in most Western countries [1, 2]. They usually include one or more visits to the doctor, during which a series of screening tests and counseling interventions are carried out. The aim of PHE is to avoid risk factors and diseases or to identify and treat them at an early stage [3–5].

The scientific evidence for the effectiveness of PHE is contradictory. Systematic reviews and large randomized controlled trials do not show any reduction in morbidity or mortality, neither overall nor for cardiovascular disease or cancer through regular screening [5–7]. In contrast, there is good evidence that individual preventive examinations lead to a reduction in risk factors that have a causal relationship with increased mortality, such as high blood pressure [5, 8]. International institutions that make recommendations for screening examinations, such as the United States Preventive Services Task Force (USPSTF) or the United Kingdom National Screening Committee (UK NSC), therefore spend a lot of time and resources weighing the benefits and harms of individual screening examinations according to evidence-based criteria.

To make sure PHE reflect high-value care, clinical effectiveness is only one aspect that needs to be taken into consideration. Equally important are the manner of implementing interventions, particularly for counseling, and the acceptance of interventions by patients. One possibility for increasing the acceptance of PHE is to involve citizens in the development of PHE recommendations. Approaches for guideline or recommendation development, e.g. the GRADE (Grading of Recommendations Assessment, Development and Evaluation) approach, aim to identify expectations, values, and preferences relating to interventions by examining the literature or consulting with individual patients and patients’ groups to assess the relative importance they place on various outcomes [9]. There is, however, a general paucity of empirical examinations of patients’ values and preferences in clinical medicine.

Not surprisingly, research evidence on PHE focusing on the patient’s or citizen’s perspective is also scarce. Comparative evidence can be derived from studies evaluating the NHS (National Health Services) Health Check, a program with the aim of reducing cardiovascular disease mortality and morbidity in the United Kingdom (UK) [10, 11]. These studies explored individual motivations for attending [10–12] and not attending health examinations [10, 11, 13, 14], as well as the experiences of patients with the NHS Health Check [12, 15, 16]. They report a number of reasons why patients attended the program, including health beliefs, the perceived value of the program [10, 12], a family history of certain diseases, expectations [12], and a desire to prevent diseases [10]. On the contrary, patients did not attend because of lack of awareness or knowledge, misunderstanding the purpose of the NHS Health Check, aversion to preventive medicine, time constraints, and difficulties with access to general practices [13]. They reported a need for individualized support in order to stay motivated and adopt long-term diet and lifestyle changes [16, 17], and often felt confused or frustrated about how results and advice were communicated [12, 15, 17]. Only one study has explored the views and experiences of patients in relation to health examinations in a setting different from the NHS Health Check [18].

As part of the update of the Austrian PHE, we explore the expectations and experiences of previous attenders of PHE. In Austria, statutory health insurances offer annual PHE to the general public. All men and women aged 18 years or older have the possibility to receive health checks for free [19]. The Austrian PHE program includes 18 screening and behavior counseling interventions (Table 1) in order to evaluate the risk of developing diseases and to prevent or detect diseases [20]. The basic examinations are offered in general practices, healthcare centres or at specialists for internal or pulmonary medicine. Some examinations, like cancer screening, are offered in specialist practices only. Patients are also referred to specials in case further examinations are required [19]. So far, the Austrian PHE program has only been evaluated from a quantitative perspective by assessing patients’ satisfaction [21]. The findings from this study will be used to design or revise the recommendations for the Austrian PHE program.

**Table 1:** Overview of the recommended preventive measures of the periodic health examination in Austria sorted by age group

Target Disease	Screening Test	Advice
From the age of 18		
Alcohol consumption	AUDIT questionnaire Determination of Gamma-GT value	Weaning advice, referral to specialized treatment for alcoholism
Diabetes mellitus	Family history, blood sugar from the vein or fingertip (First Stage Test), possible repetition of the test; oral glucose tolerance test (Second Stage)	
Glaucoma	Risk group identification with increased risk referred to ophthalmologist	
Hypertension	Blood pressure measurement (average of two measurements while sitting)	
Lipid metabolism disorders	Total cholesterol / HDL cholesterol quotient, triglyceride determination	
Malignant melanoma	Risk group identification by means of anamnesis, skin inspection of altered skin areas	Persuade patients to perform self-screening, referral to dermatologist
Periodontal disease	Questionnaire on periodontal disease, oral cavity inspection	Consultation on dental and oral hygiene, referral to periodontics-oriented dentist / physician
Tobacco / nicotine consumption	Survey of smoking status by means of the Five A’s survey	Weaning counseling (Five A’s; motivational impulses, assignment to specialized weaning facility)
Overweight / Obesity	BMI, waist circumference	Dietary recommendations, advice on physical activity supported by behavioural techniques
Cervical cancer	(Family) medical history, PAP smear	Advice on PAP smear, assignment to specialist in gynecology
(not described in detail)	Urine strip test (leucocytes, protein, glucose, nitrite, urobilinogen, blood)	
(not described in detail)	Red blood count in women	
(not described in detail)		Counseling session to motivate regular physical activity in everyday life
From the age of 45		
Breast cancer	Family history	Advice on early detection mammography
From the age of 50		
Colorectal carcinoma	FOBT, colonoscopy every 10 years	Advice on FOBT and colonoscopy, referral to medical specialist
Prostate cancer		Advice on benefits or lack of benefit or harm to PSA screening, referral to urologist / urologist
From the age of 65		
Hearing loss	Questions about hearing loss, whispering test	Referral to ENT specialist for second-stage diagnosis
Age-related visual impairment	Questions about eyesight deterioration & regular eye examination	Referral to specialist examination

*AUDIT* Alcohol Use Disorders Identification Test, *BMI* body mass index, *FOBT* Fecal Occult Blood Test, *GT* glutamyl transpeptidase, *HDL* high-density lipoprotein, *ENT* glands, nasal ears, *PAP* Papanicolaou, *PSA* Prostate Specific Antigen

Source: [20]

## Methods

### Aim

The aim of the research was to explore the motivations and reasons of adult citizens in Austria for attending PHE as well as the expectations and concerns they have in terms of their health (Aim 1). A further aim was to determine how citizens perceive the organization and process of PHE (Aim 2).

### Recruitment

We applied a stratified purposeful sampling strategy [22] to recruit previous attenders of PHE. The Health Insurance Funds of three Austrian regions made initial contact with potential participants. They sent an information package to a random sample of 1200 previous attenders of PHE, stratified by region, age, and gender. The research team selected participants who expressed interest and invited them to the focus groups based on a sampling grid that included the categories of age, gender, place of residence, education, and migration background. We applied these criteria in order to obtain diversity in the focus groups. We excluded participants if they were not able to speak German, as they were required to express themselves in a discussion held in German. The sample size of this research project was primarily guided by the heterogeneity of the target group and data saturation. To capture the diversity within each group, we aimed for a size of 8–10 participants per focus group and over-recruited by 20% to cover for no-shows.

### Data collection

We collected data using focus groups. Focus groups took place in separate meeting rooms of the Austrian Regional Health Insurance Funds. We developed a topic guide with input from the literature [11, 16, 23, 24] to elicit the discussion. Topics included reasons for attending PHE, feelings associated with PHE, dealing with results of PHE, actions upon completion of PHE, and organization of PHE. We audio-recorded the focus group discussions using a digital voice recorder. Each focus group discussion lasted up to two hours. We offered travel expenses and a small incentive (e.g. € 20 gift voucher) to all participants. We stopped collecting data after the third focus group, as no new information in relation to the research question was forthcoming.

### Data analysis

We used a thematic analysis approach [25] to inductively analyze the data. Analysis was facilitated by MAXQDA (VERBI Software.Consult.Sozialforschung GmbH). A professional transcribed all focus group discussions verbatim. We deleted audio files after transcription. After that, IS and VT conducted the data analysis. First, they read two transcripts to become familiar with the data. Then they independently identified and coded meaningful text segments. They constantly compared codes. The next step involved collating generated codes into potential subthemes. They met to review the codes and to discuss and define subthemes. The subthemes they agreed on were grouped into overarching themes. As the third focus group was conducted four months later than the first two groups, VT integrated its codes into the proposed themes and new subthemes arising. They created a graphical display that maps out the relationships of the themes. In the final step, IS selected vivid, compelling example extracts to substantiate the created themes. While she wrote the “story” of the data, she made final amendments to the themes.

## Results

From August to November 2017, we conducted three focus groups, each with 8–12 participants, in Innsbruck, Graz, and Vienna. The response rate for invitation letters sent through the Regional Health Insurance Funds was 11.4% in Innsbruck, 8.4% in Graz, and 9.7% in Vienna. The personal characteristics of participants are summarized in Table 2.

**Table 2:** Sample characteristics

	Total (*n* = 30)
Age (ø, years)	49.4
Gender (%)	
Women	46.7
Men	53.3
Place of residence (%)	
City/town	70.0
Countryside	30.0
Country of birth (%)	
Austria	100
Others	0
Nationality (%)	
Austrian	100
Others	0
Education (%)	
Compulsory school	6.7
Apprenticeship	23.3
Vocational secondary school without school leaving certificate (“Matura”)	3.3
School leaving certificate (“Matura”)	33.3
University/Polytechnic	33.3
Average frequency of periodic health examinations (%)	
Annually	66.7
Every 2 years	20.0
Every 3 to 5 years	10.0
Less than every 5 years	3.3

We identified a number of themes that were grouped into seven main themes. The first three themes were: 1) reasons for attendance, 2) reasons for non-attendance, and 3) PHE as incentive for health behavior change, relative to research aim 1. Themes four through seven were: 4) PHE should be more comprehensive and individualized, 5) PHE is a different experience with every doctor, 6) discussion of results is often dissatisfying and an emotional affair, and 7) one appointment is not enough, relative to research aim 2.

### Theme 1: Reasons for attendance

#### Health expectations

Many participants shared the expectation that PHE were all about detecting early-stage diseases or risk factors to allow for early treatment of these conditions, which would lead to a better prognosis. Attending PHE also gave participants a feeling of safety. In some cases, participants had experienced a diagnosis of severe illness as a result of PHE, which confirmed their opinion that PHE fulfilled their purpose. The early detection of diseases would prevent a lot of unnecessary suffering and would thereby make life more livable and improve well-being.

Another reason that spurred some participants to regularly attend period health examinations was the documentation of examination results. They felt that this would help establish a diagnosis or confirm that they are healthy.

#### External influences

Sometimes external influences such as sports activities requiring a medical certificate, awareness because of their healthcare profession, and the convenience of having PHE offered at work motivated participants to attend PHE. Several participants also believed that financial incentives like a bonus system or non-financial incentives, such as a working day off for attending the examination, would increase attendance rates of PHE.

#### Social influences

Family and friends but also doctors had a substantial influence on whether or not participants were attending PHE. Many participants mentioned that they are being reminded of their PHE by their doctors. Few said that PHE provided a rare occasion where doctors dedicated sufficient time, according to the patient.

In many cases, family and friends influenced participants indirectly by being diagnosed with a severe disease or suddenly dying. With others, family and friends advised or in some circumstances even pushed them to attend PHE:

*“Yes, that's the way it is for me, I'm forced to attend the PHE. My wife thinks this is important. (Note: laughs briefly) Well, my wife thinks it is important, and she is absolutely right, there are certain diseases, and the earlier they recognize them, it is about the early detection, afterwards I have a chance…” (Informant 1)*

### Theme 2: Reasons for non- attendance

Many participants believed that people often do not go to PHE for the simple reasons that they do not have it on their mind or they have other life problems that take over. Others again thought that many people would feel healthy and strong and would not see the need to attend PHE.

A few participants talked about people who were afraid of doctors and would avoid them if only possible. One participant told about an unpleasant experience he had in the past where he felt bullied with numerous examinations after revealing his smoking habits. Participants also discussed fear in terms of examinations and outcome. Some women were worried about being exposed to X-rays during mammography screening. The fear that participants believed was most decisive in not attending PHE, however, was the fear of the possible outcome and its consequences:

*“[…] if I then go a little deeper with some people, it turns out that people are afraid that something will be discovered, that's a bit like the dentist syndrome, yes, I don't go to the dentist because he could find a hole (some agree), and there I have to deal with something unpleasant.” (Informant 6)*

Another reason that many participants thought was hindering regular attendance of PHE was lack of time. PHE were generally considered to be time consuming, and many employers would not appreciate long absence periods.

### Theme 3: PHE as incentive for health behavior change

Some participants considered PHE a health-promoting activity. If a disease is detected and treated, their health would be improved and promoted. Others found themselves more aware of health issues after attending PHE. Some would actively try to change their behavior, at least if that change was easily done.

However, not all participants found recommendations for health behavior change very useful:

“*I have gone to a PHE because I was gently forced by doctors and family members, although I donate blood monthly, and the doctor recommended Nordic Walking to me, that is something that I do not like at all.” (Informant 9)*

### Theme 4: PHE should be more comprehensive and individualized

#### A range of examinations expected

The majority of participants considered the current PHE as being too simple. Many had been going to PHE for many years and observed that fewer and fewer examinations were included over time. They feared that the re-evaluation of the program would result in another downsizing of included examinations. While some thought that doctors could not offer more than a quick check because of the little money they would get, others believed exactly the opposite:

*“[…] on the other hand, I find it is a cheaply earned money for the doctor, I must say quite honestly, there is simply too little in the whole package, it just should be fundamentally, the package should be extended.” (Informant 5)*

When asked which examinations should be included in PHE, participants listed a number of examinations ranging from orthopedic examinations to lifestyle consultation. Some thought that PHE would no longer be up to date to meet present health problems. Others took the view that patients should have a say in which examinations the doctor should conduct or arrange.

#### Examinations should be selected based on individual risk

Although many participants wanted PHE to include a full spectrum of examinations, they considered a differentiation according to age and family history as important. Older people as well as younger people with family history would require more in-depth examinations.

### Theme 5: PHE a different experience with every doctor

Many participants felt that doctors would have to deal with an increasing number of patients and a growing bureaucracy these days, giving them less and less time to spend with the individual patient during PHE. However, there were some exceptions. In some cases, assistants conducted the PHE, with patients only seeing the doctor for the discussion of the results.

Throughout the discussion, participants realized there was a great diversity in the content of PHE they received:

“*May I tell you something, I think every doctor is really different and some doctors can, can just put that in and some can't. The form, which they send to the laboratory is not predefined, the doctor can still add whatever he likes.” (Informant 9)*

Choosing the right physician was also important to participants. A number of participants expressed their frustration with the statutory health insurance physicians, particularly those practicing in urban areas, because of the little time they dedicated to their patients. These participants preferred private physicians. When patients go to private physicians, they are reimbursed part of the costs by the health insurance fund. Likewise, many would see the specialist for their PHE. Internists would have better equipment, could fit in more examinations, and therefore would save patients time and extra doctor visits.

### Theme 6: Discussion of results is often dissatisfying and an emotional affair

Many participants were unhappy with the way doctors discussed the results of the PHE. In their view, doctors did not realize or care if patients understood what the results meant and which consequences they carry. The most important problem was the use of medical terms:

“*He can't bring it across so that us, I just say it using quotation marks, ordinary citizen could understand it, because he comes with his technical jargon, I say it provocatively now, because I cannot make use of all those words, we can't do anything with them or at least I can't […].” (Informant 22)*

In addition to communication problems, many participants wished doctors would spend more time discussing the results. They often felt left alone with borderline findings and had the feeling that whether or not they would be given advice was totally dependent on the doctors.

Participants described a series of feelings they had in anticipation during or after the discussion of the results of PHE. These feelings ranged from shock having received a positive test result to being disappointed because their effort to change their lifestyle was unsuccessful.

### Theme 7: One appointment is not enough

Some participants suggested developing goals for change with their doctors as result of a PHE. These goals should be evaluated in follow-up appointments or in the next PHE at the latest.

“*Yes, I also think that somehow the patients' personal responsibility should somehow be focused in this way, that one really should agree on concrete goals, and then come back again and have another conversation and perhaps also work with some incentive systems [...]” (Informant 28)*

Participants also mentioned they would appreciate it if doctors reminded them of follow-up examinations or further examinations with specialists that were due.

In terms of further examinations with specialists, many participants expected the PHE with the general practitioner to be only the starting point of further examinations. However, doctors would again behave very differently, and not all would be willing to prescribe further examinations.

Apart from that, participants experienced further examinations as cumbersome because of the extra doctor visits. They would find it helpful if doctors collaborated with specialists. Additionally, further examinations would sometimes not be covered by health insurance, which they felt was unfair. Although others were also not happy having to pay for further examinations, they viewed them as extra service doctors were offering.

## Discussion

### Summary of main findings

Our study found that the main motivation for people to attend PHE was to detect and treat diseases early and to live a long and healthy life. Alongside health expectations, other individual, social, or external factors were decisive for attendance. When asked about possible reasons for non-attendance, participants mentioned lack of awareness, time constraints, unpleasant experiences, and fear of harm or outcome.

Participants had high expectations of PHE. In the view of most, PHE in its current form was too simple and should include a range of examinations tailored to individual risks and needs. Some called for standardization of the procedure of PHE and blamed the health system for the time and financial constraints that doctors face. Other aspects of improvements included the final discussion of results and arrangement of follow-up or further examinations.

### Comparison with existing literature

Several studies report similar reasons for attending and non-attending PHE, thereby confirming our findings. The desire to prevent and detect diseases early, reassurance about health, and reinforcement of healthy lifestyles also motivated patients to attend the NHS Health Check [10–12]. Likewise, social factors influencing uptake of screening programs, such as the obligation toward family and friends or ill health of those, was described not only by patients [11, 12, 18, 23] but also by general practitioners offering health examinations [24]. The latter finding suggests that a range of approaches (e.g. integration of family members) are to be realized in order to increase the uptake among those that are not motivated from the outset.

Reasons for non-attendance found in our study also resonate with previous publications. A qualitative review on reasons for non-attendance of the NHS Health Check identified lack of awareness or knowledge, avoidance of practices because of previous experiences or when feeling well, time constraints, or competing priorities as barriers [13]. Burgess et al. 2014 [11] further reported concerns about negative consequences of having a health examination, which our participants discussed as fear of outcome. Therefore, doctors should communicate health information in a non-threatening way that does not fuel fear among patients.

Participants also expressed fear in relation to harms of screening tests but not harms of follow-up testing and treatment. This is in line with a study conducted in the Unites States where patients could easily identify benefits of screening while struggling to identify harms other than those of screening tests [26]. The authors explained their finding with a lack of information on possible harms that is given to the patients, which could also be the case in our context.

Many participants were not pleased with the advice and support given to make lifestyle changes and anticipated that PHE would continue with follow-up appointments or referrals. Similarly, they considered the time and effort doctors took to discuss the results as too short, and they would often struggle to understand the implications. These experiences are concordant with those of patients attending the NHS Health Check [17] . A Cochrane Review from 2012 showed that training providers of healthcare (even less than 10 h) has positive effects on consultation processes on a range of measures relating to clarifying patients’ concerns and beliefs [27]. Such training might also be useful to doctors providing PHE in Austria. Additionally, they should set and follow-up goals for long-term diet and lifestyle change.

Expectations of PHE in terms of comprehensiveness and individualization by the participants in our study were high. In their view, the current PHE was far too simple and should include a range of examinations based on an individual’s risk. This finding is contradictory to the lack of scientific evidence regarding the effectiveness of PHE [5–7]. It seems that patients believe that the more examinations they receive the better for their health. This reflects serious shortcomings in the communication of benefits and harms of screening interventions. In addition to trainings for healthcare providers, existing information campaign on benefits and harms of individual examinations of PHE targeting patients should be revised or extended.

The request of participants to standardize the content of the PHE after realizing that each PHE was conducted differently is confirmed by the study of Sondergaard et al. 2014 [24], which revealed a great diversity in content and quality of health examinations. Some participants explained the diversity to some extent with the type of contract and specialization of doctors offering PHE, which needs further investigation. Nonetheless, to increase standardization, it might be helpful to revise the current Austrian guideline for PHE [20] and evaluate its implementation. The information campaign should further clearly communicate the content of the PHE as covered by the health insurance funds.

### Strengths and limitations of the study

The strengths of this study include the acquisition of participants through the databases of the Regional Health Insurance Funds. We could thereby reach a high number of participants varying in age, gender, place of residence, and education.

Another strength is the dual involvement of the investigators IS and VT in the data collection and analysis process. This approach enabled us to reflect and discuss the focus groups. IS and VT both have a non-medical background with little preconceptions of the practice of PHE, which has made it easier to create themes that were fully emerging from the data. Nonetheless, we are individuals with our own ideas and belief systems that have naturally had an influence on the data.

Limitations of this study are also recognized. We only recruited attenders of PHE, so motivations and reasons for non-attendance that we derived are based on speculations made by attenders rather than firsthand information from non-attenders.

Another limitation refers to the failure to acquire participants with a migration background. In 2016, the proportion of Austrian citizens who were born abroad was 16% [28]. Despite the large number of insured adults we contacted, we had no interest from people with migration background in taking part in the focus groups. One reason for that could be language barriers as we required participants to take part in a discussion in German. We do not know whether their perceptions of the PHE are similar to our participants or not, and can therefore not extend our findings to this group.

In terms of size of each group, we ended up with 8–12 participants due to 20% over-recruitment and considerations of heterogeneity. It is possible that the relatively large group sizes have limited the individual’s opportunities to share experiences and insights. We did, however, do our best to engage all participants into the discussion and gave them sufficient time for it (up to two hours). Additionally, there is no agreement within the qualitative methods literature on ideal focus group size, with recommendations ranging from 4 to 12 participants [29].

Finally, we did not return transcripts to participants or seek their feedback on themes derived in this study (member checking). Although this is often assumed to improve the credibility of qualitative research, it would have been difficult for individual participants to identify their statements in the transcript. In addition, a recent study on the impact of member checking found little evidence that member checking improved research findings [30].

## Conclusions

Our study has identified a number of reasons why adult citizens attend or do not attend PHE. Expectations for the content and quality of PHE were high, with a request for a more comprehensive and individualized program that is standardized in its procedure. These expectations do not reflect findings of scientific studies about the effectiveness of PHE, but rather indicate serious shortcomings in the communication of benefits and harms of screening interventions. The findings highlight the need for a more proactive communication about aims and content of the program which could be achieved by a revised or extended information campaign targeting patients, an update of the current best practice guideline of the Austrian PHE, and training healthcare providers in patient-centered communication. Further studies are needed to explore motivations and expectations for PHE among migrant groups.

## Abbreviation

PHE: Periodic health examination/s
